# Myeloid Derived Suppressor Cells Migrate in Response to Flow and Lymphatic Endothelial Cell Interaction in the Breast Tumor Microenvironment

**DOI:** 10.3390/cancers14123008

**Published:** 2022-06-18

**Authors:** LaDeidra Monét Roberts, Matthew J. Perez, Kristen N. Balogh, Garnett Mingledorff, Janet V. Cross, Jennifer M. Munson

**Affiliations:** 1 Department of Biomedical Engineering and Mechanics, Fralin Biomedical Research Institute, Virginia Tech, Roanoke, VA 24016, USA; monetr@vt.edu; 2 Department of Biomedical Engineering, University of Virginia, Charlottesville, VA 22904, USA; mjp2tv@virginia.edu; 3 Department of Pathology, University of Virginia, Charlottesville, VA 22904, USA; knr4mc@virginia.edu (K.N.B.); jvc5b@virginia.edu (J.V.C.); 4 Department of Microbiology, Immunology, and Cancer Biology, University of Virginia, Charlottesville, VA 22904, USA; gam7t@virginia.edu

**Keywords:** myeloid derived suppressor cells, cancer cell migration, interstitial fluid flow, lymphatic endothelial cells, tumor microenvironment, 3D model, VEGFR3, CXCR4, MIF, breast cancer

## Abstract

**Simple Summary:**

Myeloid-derived suppressor cells (MDSCs) are a subset of immune cells that contribute to a pro-tumorigenic microenvironment, promoting immunosuppression in multiple tumors. In breast cancer, they have been mostly studied in pre-clinical mouse models. These models allow for examination of the development of pre-metastatic niches and immune infiltration at the primary tumor site, but are difficult to use to extricate complex intercellular signaling events and MDSC migration. Here, we describe the development of a breast tumor microenvironment model that includes primary-derived mouse MDSCs. We use this model to identify interactions that drive MDSC migration through both biophysical and cellular signaling events within the tumor microenvironment.

**Abstract:**

At the site of the tumor, myeloid derived suppressor cells (MDSCs) infiltrate and interact with elements of the tumor microenvironment in complex ways. Within the invading tumor, MDSCs are exposed to interstitial fluid flow (IFF) that exists within the chronic inflammatory tumor microenvironment at the tumor–lymphatic interface. As drivers of cell migration and invasion, the link between interstitial fluid flow, lymphatics, and MDSCs have not been clearly established. Here, we hypothesized that interstitial fluid flow and cells within the breast tumor microenvironment modulate migration of MDSCs. We developed a novel 3D model to mimic the breast tumor microenvironment and incorporated MDSCs harvested from 4T1-tumor bearing mice. Using live imaging, we found that sorted GR1+ splenocytes had reduced chemotactic index compared to the unsorted population, but their speed and displacement were similar. Using our adapted tissue culture insert assay, we show that interstitial fluid flow promotes MDSC invasion, regardless of absence or presence of tumor cells. Coordinating with lymphatic endothelial cells, interstitial fluid flow further enhanced invasion of MDSCs in the presence of 4T1 cells. We also show that VEGFR3 inhibition reduced both MDSC and 4T1 flow response. Together, these findings indicate a key role of interstitial fluid flow in MDSC migration as well as describe a tool to explore the immune microenvironment in breast cancer.

## 1. Introduction

Breast cancer is one of the most commonly diagnosed cancers worldwide [1]. Myeloid-derived suppressor cells (MDSCs) drive, in part, an immunosuppressive tumor microenvironment which contributes to worsened disease. They are a heterogeneous population of immature myeloid cells that are recruited to and aid in formation of the pre-metastatic niche through tumor propagation, dissemination, and seeding by suppressing antitumor immune regulation [2,3,4]. Higher MDSC levels have been associated with metastatic breast cancer within the tumor, circulation, and metastatic sites, thereby correlating to poor patient prognosis [5]. First discovered in cancer, MDSCs have more recently been connected to other pathological states in rodent models, including inflammation, wound healing, transplant immunity, infection [6], and vaccination efficacy [7].

Most MDSC studies have been confined by in vivo studies wherein MDSCs are examined within the circulation, primary tumor site, spleen, or potential sites of metastasis. Although they are best characterized in mice, markers for the identification of MDSCs in other species remain controversial [8]. Across studies, different biomarkers and specific subtypes of MDSCs are involved in various locations in the body as well as physiological and pathological states, all contributing to a lack of consensus around characterization, especially in human patients. Advances have been made demonstrating different biomarkers, such as surface markers, secreted cytokines and growth factors, and MDSC-derived exosomes, in efforts to demystify their elusive and variable characterization and roles in pathology and physiology; however, there is still a need for standardization in the field [2,9,10,11,12,13]. Nonetheless, techniques to study these cells in vivo have been fairly well-established in mice.

In cancer, MDSCs are known contributors to the reorganization of the extracellular matrix (ECM) towards a pro-tumorigenic and metastatic state. Due to their immature myeloid signature and array of phenotypes, they coordinate with extracellular cues, such as ECM modification and chemotactic signaling [14,15]. Thus, research suggests that the tumor microenvironment plays an important role in influencing MDSCs in tumor progression [16,17,18,19,20]. Most studies focusing on MDSCs are in vivo, due to the need for an active tumor to generate the cells. In vitro models have focused on proliferation, suppressive activity, migration, and tumor microenvironment actors in 2D [21,22,23]. One recent study discovered stromal hepatic stellate cells influenced MDSC migration both in vitro in 2D and in vivo [23]. However, these in vitro models have not incorporated the ECM in a three-dimensional context, which is important to consider in physiologically recapitulating cellular interactions and extracellular cues in the tumor microenvironment.

Elevated peripheral interstitial fluid flow (IFF), due to increased drainage from the tumor, towards collecting lymphatics, is a driver of cellular activation, invasion, and immune trafficking. The lymph nodes, to which the lymphatics drain, serve as a terminal site for flow and MDSC mobilization [18,24,25,26]. IFF, lymphatics, and draining lymph nodes are all modulators of the immune microenvironment of tumors, often promoting immunosuppression in line with that seen with MDSCs. How IFF and lymphatic endothelial cells interact with MDSCs to promote migration is not clearly established. In vitro models offer a useful tool to probe the individual and combinatorial effects of the components of the tumor microenvironment as well as any tissue engineered microenvironment. Here, we show the development of an in vitro 3D tissue culture model in which to study MDSCs and describe their migration characteristics alone as well as in the presence of other key players in the tumor microenvironment. Through these studies, we provide a template for how MDSCs might be studied in a physiologically relevant manner in vitro.

## 2. Materials and Methods

### 2.1. Mice

Female 6- to 12-week-old BALB/c mice were purchased from Charles River Laboratories. All animal experiments were performed with the approval of the University of Virginia Animal Care and Use Committee (ACUC).

### 2.2. Cell Culture

Immortalized mouse lymphatic endothelial cells (iLECs) were cultured as previously described [27]. In brief, iLECs were cultured in 40% DMEM/40% F12 with 20% fetal calf serum, 56 μg/mL heparin, 10 μg/mL endothelial cell mitogen, and 1 U/mL mouse IFNγ at 33 °C until 70–80% confluence. Cells were then passaged and cultured at 37 °C without IFNγ to induce differentiation towards the LEC phenotype. Mouse mammary carcinoma cell line 4T1-luc-red were purchased by the Cross lab from Perkin-Elmer (BW124087V) after lentiviral transduction of the Red-FLuc luciferase gene. Additionally, 4T1-luc-red cells were cultured in RPMI medium supplemented with 10% FBS. All cell lines were grown under sterile conditions in a humidified atmosphere of 5% CO_2_ and 95% oxygen at 37 °C.

### 2.3. Splenocyte Isolation and Sorting

In keeping with prior methods for MDSC generation and isolation from the spleen [28,29,30], 4T1 tumor bearing mice were used as a source of MDSCs and other system components. Three weeks (21 days) after implantation of 10,000 4T1 cells into the mammary fat pad of a Balb/c mouse, abundant MDSCs (CD11b+, GR1+ cells) of very high purity were isolated from the spleen by magnetic bead isolation. These cells are the gold-standard of MDSCs and best representative of the cells that traffic to the niche in vivo. Therefore, they were used for the establishment and validation of the murine model. Splenocytes were then sorted into GR1+ and GR1− populations by immunomagnetic separation using the EasySep^TM^ Mouse PE Positive Selection Kit (STEMCELL Technologies, #18554, Vancouver, Canada) and GR1 PE antibody (Biolegend, #108408, San Diego, CA, USA). To ensure that the murine cells exhibited similar properties regardless of the protocol used to prepare them, we produced murine MDSCs by culturing mouse bone marrow in a similar cytokine regimen to human cells [31]. All MDSC populations were confirmed to have the characteristic “suppressive activity” using T cell suppression assays established in our lab [30].

### 2.4. Three-Dimensional Cell Culture Model

A hydrogel comprised of 1.8 mg/mL rat tail collagen I (Corning) and 0.5 mg/mL growth factor-reduced basement membrane extract (Trevigen) was used to model the breast tumor microenvironment. Then, 100,000 4T1-luc-red cells and/or 100,000 splenocytes were seeded in the gel prior to polymerization. The cell–gel suspension was seeded in either a 96-well plate for time-lapse microscopy or a transwell for invasion assay. After cells were added, the gels polymerized in a humidified incubator (5% CO_2_, 95% oxygen, 37 °C) for 30 min, yielding a 100 µm-thick gel as previously described and characterized [32]. After polymerization, serum free RPMI containing 10 ng/mL granulocyte-macrophage colony stimulating factor (GM-CSF) was applied (R&D, #415). For static conditions, 100 µL of media was applied atop the gel with 700 µL in the exterior compartment. For flow conditions, 700 µL was applied atop the gel with 100 µL underneath the tissue culture insert. This would cause a slow flow through the gel at a rate of approximately 1 µm/s as measured by volume displacement.

### 2.5. Time-Lapse Microscopy

First, 4T1s, MDSCs, or both were seeded in 100 µL gels in a 96-well plate and allowed to polymerize for 30 min, flipping the plate every 5 min to prevent cells from settling. Time-lapse microscopy was performed using an EVOS FL Auto or an EVOS FL Auto 2.0 with an incubated stage (Thermo Fisher, Waltham, MA, USA). Locations in each well were selected using the EVOS software, and each location was imaged every 5 min for 18 h.

### 2.6. Cell Tracking

Image stacks from time-lapse microscopy were used for cell tracking. Cells were tracked individually using the manual tracking plugin in ImageJ. Results from the manual tracking program (slice number, x-coordinates, y-coordinates) were imported into Matlab and analyzed using a previously published script to calculate mean speed, total displacement, and chemotactic index [32,33]. The percent of migrating cells was determined manually by counting the total number of cells in a field of view and the total number of cells migrating more than two cell body lengths from their original position. These numbers were divided to determine the percentage of cells migrating within a field of view.

### 2.7. Invasion Assay

To begin, 100,000 4T1 cells and/or 100,000 MDSCs were added to the collagen–BME hydrogel and seeded atop an 8 µm pore tissue culture insert (Millipore, Burlington, MA, USA). Before incorporation into the gel, MDSCs were labeled with Cell Tracker Green (Thermo Fisher, Waltham, MA, USA). For studies involving iLECs, 150,000 iLECs were seeded on the underside of the transwell membrane prior to seeding the gel inside of the transwell. For inhibition studies, 10 μM AMD3100, a selective CXCR4 antagonist, 2 μM Sulforaphane (SFN), a macrophage migration inhibitory factor (MIF) inhibitor, 1 μM MAZ51, a VEGFR3 antagonist, or DMSO as vehicle were added to all media and gel. The gel was exposed to static and flow conditions for 18 h, at which point invasion was measured.

### 2.8. Invasion Analysis

Media was collected from the bottom of the tissue culture insert and the MDSC concentration was measured by flow cytometry. The bottom of the tissue culture insert was also fixed and imaged using fluorescence microscopy. Then, the number of MDSCs on the underside of the tissue culture insert was counted, where the total number of invaded cells was used to calculate the percentage of invaded cells (% invasion = number on insert bottom/number seeded × 100).

### 2.9. Statistical Analysis

Two-way ANOVA was performed for groups of two or more, with a *p*-value < 0.05 indicating statistical significance. When significance was observed, ad hoc *t*-tests were performed to assess statistical significance between specific groups. Either a ratio paired or unpaired student’s *t*-test was performed, with *p*-value < 0.05 considered as significant. All graph data are represented as mean ± standard error mean. For data that were represented in histograms for migration outcomes, statistical significance was determined by Kolmogorov–Smirnov test, with *p*-value < 0.05 considered as significant. Individual statistical assessments are discussed with the presented data.

## 3. Results

### 3.1. Development of a 3D Culture Model for MDSCs

**Figure 1:**
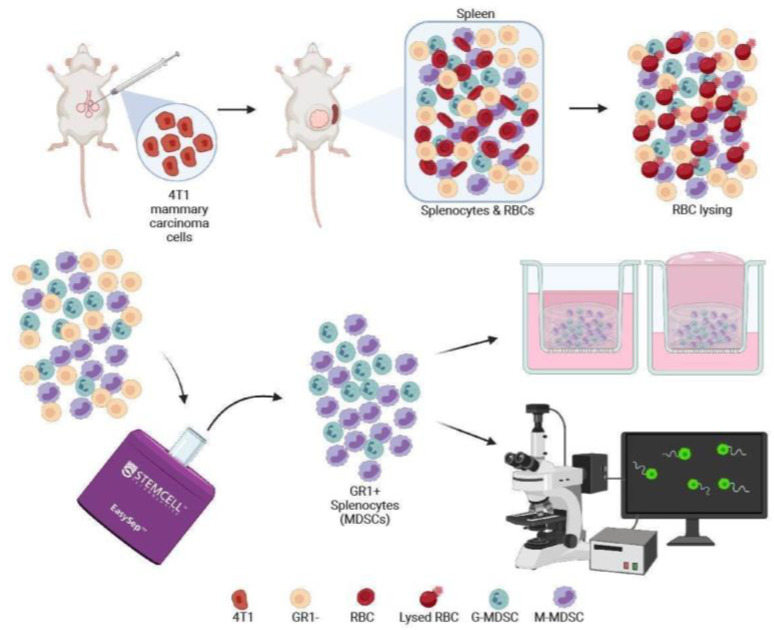
Workflow schematic for harvesting MDSCs for analysis of invasion and motility. 4T1 murine mammary carcinoma cells were injected into a female Balb/c mouse. When tumor size was sufficient, the spleen was harvested and digested for splenocyte isolation and EasySep^TM^ immunomagnetic sorting. MDSCs then underwent further testing in 3D transwell assays to assess invasion or live imaging for cell tracking. (Created with BioRender.com. Accessed on 1 August 2021.)

To generate and harvest MDSCs in line with previously published methods [30], we injected the murine 4T1-luc-red breast cancer cell line into mammary fat pads of mice (Figure 1). After sufficient tumor growth, the spleen becomes one of the major sites for MDSC expansion. Therefore, spleens from tumor-bearing mice were harvested and underwent RBC lysing to remove red blood cells before GR1+ and GR1− immunomagnetic sorting to isolate a population of majority MDSCs. To understand MDSC migration in the tumor microenvironment of breast cancer, we then developed a cell culture model of the breast tumor microenvironment to culture MDSCs in 3D. For a holistic approach for assessing MDSC migration, cells were added to collagen gels for either live imaging using time lapse microscopy of cell motility or seeded into tissue culture inserts to mimic the breast tumor microenvironment [34] to assess invasion.

### 3.2. GR1+ Cells Sorted from Splenocytes have Reduced Overall Migration

**Figure 2:**
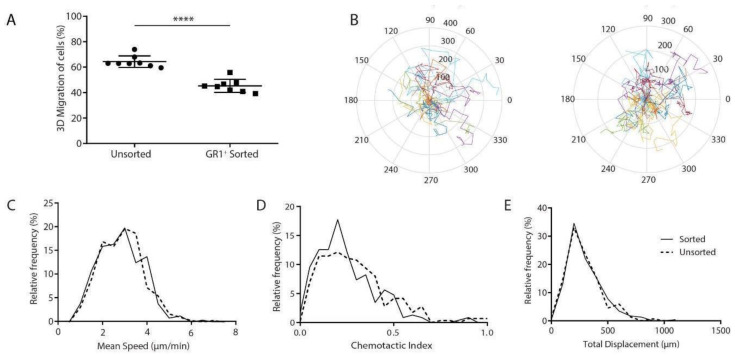
GR1+ cells sorted from splenocytes have reduced overall migration. (**A**) Quantification of unsorted and sorted splenocyte percent migration in our 3D in vitro model (*n* = 4; bars and error represent mean ± SEM, ****, *p* < 0.005). (**B**) Representative polar plots of migration tracks of unsorted and sorted splenocytes through live imaging. (**C**–**E**) Histograms of mean, chemotactic index, and displacement of unsorted (dashed line) and sorted (solid line) cells across live imaging experiments. All data in histograms represented across live imaging experiments with statistical significance were confirmed in the chemotactic index between the unsorted and sorted population of GR1^+^ cells through the Kolmogorov–Smirnov test (*p* < 0.05).

We first used live imaging and tracking of sorted and unsorted splenocytes alone in our 3D collagen gels to determine whether there was an impact of the sorting process on cellular motility. GR1+ enriched splenocytes showed fewer migrating cells as determined by calculating the overall percent migration compared to the unsorted population of splenocytes (Figure 2A). The migrating cells were tracked to quantify the migration characteristics of speed, displacement, and chemotactic index. Migrating cell tracks indicated similar distributions and lengths when comparing GR1+ splenocytes to the unsorted splenocytes (Figure 2B). Quantification of these tracks indicated that migrating GR1+ sorted cells had the same speed and displacement as the unsorted population (Figure 2C–E). However, GR1+ sorted cells had significantly decreased chemotactic index compared to the unsorted population (Figure 2C). These results suggest that sorting results in a population with fewer migrating cells, however, the cells that are migrating have similar properties according to the motility metrics that we assessed.

### 3.3. Migration Characteristics of MDSCs Are Sensitive to Harvest

It has been previously established that MDSC function can be dependent on various factors within the tissue as well as methodological techniques in isolation and cryo-preservation [12,35,36]. In harvesting MDSCs, magnetic beads were used to separate the GR1+ population from the unsorted population (Supplementary Figure S1). We saw that both the presence of the beads for separation and the actual subpopulation of GR1+ cells resulted in reduced percent migrating cells, indicating a potential combinatorial effect of the harvest with the biological subpopulation (Supplementary Figure S1A). However, the speed, chemotactic index, and displacement of these populations were again similar (Supplementary Figure S1B). Since MDSCs must be freshly harvested from tumor-bearing mice, we wanted to examine migration differences between populations isolated from different mice under independent inoculation and harvest dates. Between mice, the percentages of migration slightly varied, though they were similar. This suggests that MDSC function, specifically migration, can be sensitive to the current methods of generation and isolation (Supplementary Figure S2). More importantly, this limitation would not necessarily only apply to our in vitro assays, since this is also similar to how experiments are performed in vivo with MDSCs. We determined that since migration characteristics were similar, even though the overall number of migrating cells was reduced, we would move forward with the GR1+ sorted population to best ensure a more pure population. For the sake of clarity, this GR1+ population will be referred to as MDSCs throughout the subsequent experiments.

### 3.4. MDSCs Migrate Differently in the Presence of Tumor Cells

**Figure 3:**
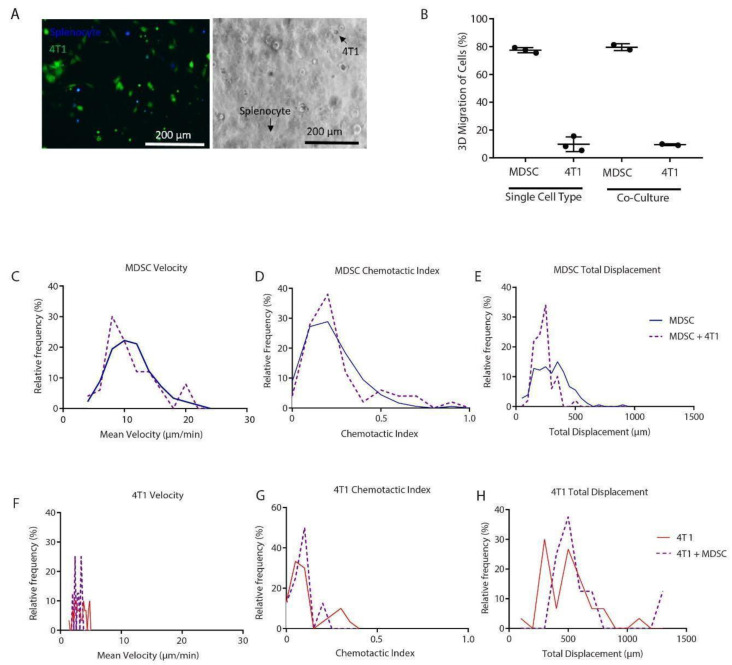
MDSCs migrate at higher frequency and faster than 4T1 cells in co-culture. (**A**) Representative fluorescent images of sorted GR1+ MDSCs and 4T1 tumor cells in 3D co-culture (splenocytes, blue; 4T1: green) (left) and brightfield (right; black arrows indicate specific cells of interest) (scale bar: 200 µm). (**B**) Quantification of percent migration of MDSCs and 4T1 alone and in co-culture in the 3D in vitro model (*n* = 3, single cell type and *n* = 2 for co-culture; data represent mean ± SEM). (**C**–**E**) Histograms of mean, chemotactic index, and displacement of MDSCs alone (blue solid line) and MDSCs + 4T1 cells (purple dashed line). (**F**–**H**) Histograms of mean, chemotactic index, and displacement of 4T1s alone (red solid line) and 4T1 cells + MDSCs (purple dashed line). All data across live imaging experiments are represented as histograms.

Tumor cells have been implicated in promoting MDSC expansion and function through secretion of tumor-derived factors [36]. In our 3D culture model, we wanted to observe MDSC motility in co-culture with 4T1 breast cancer cells by examining their migration individually and collectively (Figure 3A). In the presence of 4T1 cells in co-culture, there was no significant difference in the percentage of MDSCs migrating. However, this assay highlighted that MDSC and 4T1 migration can be measured simultaneously (Figure 3B). Although the percent migration of MDSCs was not affected by tumor cells (Figure 3B), MDSCs had a much higher population of migrating cells than tumor cells. MDSC migration speed (Figure 3C) was much greater than 4T1 migration speed (Figure 3F). Total displacement of MDSCs was reduced in the presence of 4T1 cells (Figure 3E,H), which is especially important since tumor cells migrate slower in comparison to immune cells. Moreover, MDSC chemotactic index was increased with the addition of 4T1 cells (Figure 3D,G), most likely due to the chemotactic signaling of tumor cells with the MDSCs. Overall, these results suggested that increased migration may be promoted by the interplay of MDSCs with tumor cells.

### 3.5. MDSCs Respond to Both Interstitial Fluid Flow and Lymphatic Endothelial Cells by Increasing Their Migration

**Figure 4:**
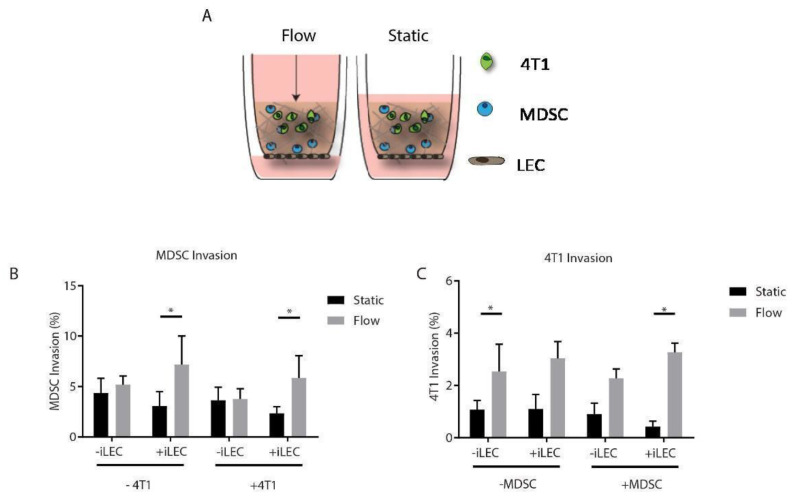
Lymphatic endothelial cells and flow coordinate in enhancing migration of MDSCs and 4T1s. (**A**) Schematic of 3D cell culture model with collagen−BME hydrogel and 4T1s, MDSCs, and LECs under flow (left) and static (right) conditions. (**B**) Quantification of GR1+ MDSC percent migration in the presence of both iLECs and 4T1 cells under static and flow conditions (*n* = 3). (**C**) Quantification of 4T1 percent migration in the presence of iLECs and MDSCs (*n* = 3). Data and error bars represent mean ± SEM (*, *p* < 0.05).

Along with the spleen, lymph nodes are a common site for MDSC accumulation in cancer [4]. MDSCs, particularly of the monocytic phenotype, have been shown to home to tumor-draining lymph nodes in the setting of thymus, breast, and lung cancer in mice [25]. The lymphatic-associated growth factor, VEGF-C, has been proposed to promote recruitment of MDSCs [24]. Furthermore, we and others [37] have previously shown that there is crosstalk between the tumor and lymphatics in the breast tumor microenvironment, and this interaction promotes migration of breast tumor cells in vitro [34]. How MDSC migration is impacted in the presence of lymphatic endothelial cells (LECs) in breast cancer has not yet been established. Therefore, we incorporated murine LECs (iLECs) along the tissue culture insert bottom as a monolayer to simulate a draining lymphatic vessel. We then established a 3D co-culture of 4T1 cells and MDSCs in the same collagen gel system used for live imaging to physiologically mimic the tumor microenvironment (Figure 4A). We then assessed the invasion of 4T1 cells and MDSCs under static and flow conditions alone and together (Figure 4B,C). Interestingly, we did not see a baseline increase in the invasion of any cell type in static conditions. MDSCs increased their invasion in the presence of iLECs and interstitial flow together, but not either cell component alone (Figure 4B). The presence of 4T1 cells did not impact MDSC invasion (Figure 4B). In general, 4T1 cells invaded more with flow, and significant invasion was seen only in 4T1s alone and when both MDSCs and iLECs were present with flow, suggesting a potential microenvironmental interaction (Figure 4C). These results indicated that lymphatics and flow together stimulate MDSC invasion. Moreover, for 4T1 cells, invasion is increased by interstitial flow in a cellular context dependent manner.

### 3.6. Inhibition of VEGFR3 Blocks Migration of MDSCs and 4T1s

Since we observed that there were complex interactions within the 3D model leading to increased invasion under flow (Figure 4B,C), we were interested in observing whether inhibition of known mediators of flow-mediated cellular invasion and activation could negate these effects. MDSC and tumor cell migration have been linked to complex intercellular signaling events that can be mediated by a number of chemokines and receptors. Therefore, we used pharmacological inhibitors to block specific molecular players involved in MDSC–tumor cell interactions. CXCR4 has been implicated in the migration of 4T1 tumor cells, resulting in systemic metastasis to lymph nodes through chemotactic signaling from ligands expressed by LECs [37,38,39]. Lymphangiogenic receptor VEGFR3 has also been proposed to be a key molecular player in cancer cell–lymphatic crosstalk, specifically through its associated ligand VEGF-C, which is linked to poor prognosis in breast cancer [24,34]. Both receptors have also been implicated in flow-mediated migration and activation. Macrophage migration inhibitory factor (MIF) is a mediator of MDSC migration and proliferation in the promotion of tumor growth and metastasis [30,40,41]. Therefore, we blocked MIF using the known inhibitor Sulforaphane (SFN) [30,40,42]. Certain surface molecules on cancer cells, such as CXCR4, have been shown to be involved in interstitial flow-mediated invasion in various cancers [32,43,44,45]. Therefore, we used the selective CXCR4 antagonist AMD3100. Lastly, to target LECs with known fluid-mediated activation of VEGFR3, we used the targeted inhibitor MAZ51 [46,47]. We were primarily interested in the changes to invasion seen within the model comprised of all cell types, since we had seen significantly increased invasion of both MDSCs and 4T1s in this context.

**Figure 5:**
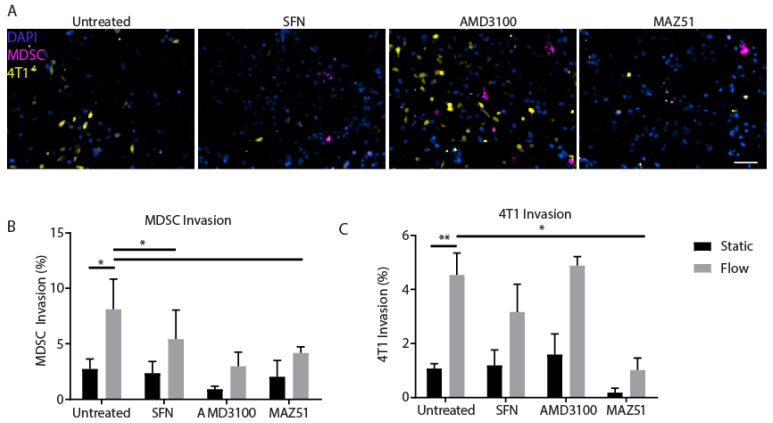
Inhibition of VEGFR3 significantly decreases fluid-mediated invasion of 4T1s and MDSCs in the 3D tumor microenvironment co-culture model. Co-cultures of MDSCs, 4T1s, and LECs were treated with three inhibitors or untreated (DMSO) in tissue culture inserts. (**A**) Representative images of invading cells on tissue culture insert membranes for each inhibitor. Scale bar represents 200 µm. (**B**) Quantification of MDSC invasion in the presence of 2 µM SFN, 10 µM AMD3100, and 1 µM MAZ51 under static and flow conditions (*n* = 3). (**C**) Quantification of 4T1 invasion in the presence of 2 µM SFN, 10 µM AMD3100, and 1 µM MAZ51 under static and flow conditions (*n* = 3). Data and error bars are represented as mean ± SEM, *, *p* < 0.05 and ** *p* < 0.01.

As seen in the prior experiment (Figure 4), the combination of all cell types with flow enhanced invasion of both MDSCs and 4T1s (Figure 5). MIF inhibition significantly reduced invasion of MDSCs towards iLECs under flow but did not reduce migration generally (Figure 5B). Sulforaphane had no effect on 4T1 invasion (Figure 5C). Surprisingly, inhibition of CXCR4 with AMD3100 did not inhibit invasion of 4T1s either. Both cell types, MDSCs and 4T1s, had reduced invasion towards the iLEC monolayer when VEGFR3 was inhibited with MAZ51. Inhibition of any target under static conditions did not significantly reduce invasion of MDSCs nor 4T1s. These results support the role of VEGFR3 as a potential mediator of fluid flow-enhanced migration of both 4T1s and MDSCs in the context of the tumor microenvironment.

## 4. Discussion

MDSCs are both affected by and alter the tumor microenvironment. MDSCs result from the constitutive pro-inflammatory cytokines and chemokines from the tumor [4,48]. This, in turn, causes MDSCs to suppress their innate progenitor immune cell counterparts as well as components of the adaptive response and prevent antitumor immune response [4,35,49,50]. An important step in the function of MDSCs is mobilizing to the tumor site by way of the bloodstream and peripheral lymphoid organs [3,51]. However, direct mechanisms involved in mobilization are not fully elucidated. Within the tumor microenvironment, enhanced interstitial pressure in the tumor promotes increased interstitial fluid flow, thereby increasing drainage and transport of molecules [18,26,52]. In breast cancer, including triple negative breast cancer, MDSCs have been implicated in aggressive phenotypes and promoting metastasis [5,53,54]. Moreover, coordination with the lymphatic system through fluid flow and drainage has been established as important in immunity and promoting immunosuppressive microenvironments [18,19,26,34,55]. Interstitial fluid flow works to transport cytokines through the tumor site and into draining lymphatics, acting as a mediator of immune cell trafficking [56], suggesting that it could play a similar role in MDSC trafficking and infiltration. Although there have been studies focused on migration and recruitment of MDSCs in 2D [20,21,22,23,24], we hypothesized that increased interstitial fluid flow would enhance migration of MDSCs and, in coordination with other tumor microenvironment actors, alter physiological responses in a 3D model of breast cancer.

**Figure 6:**
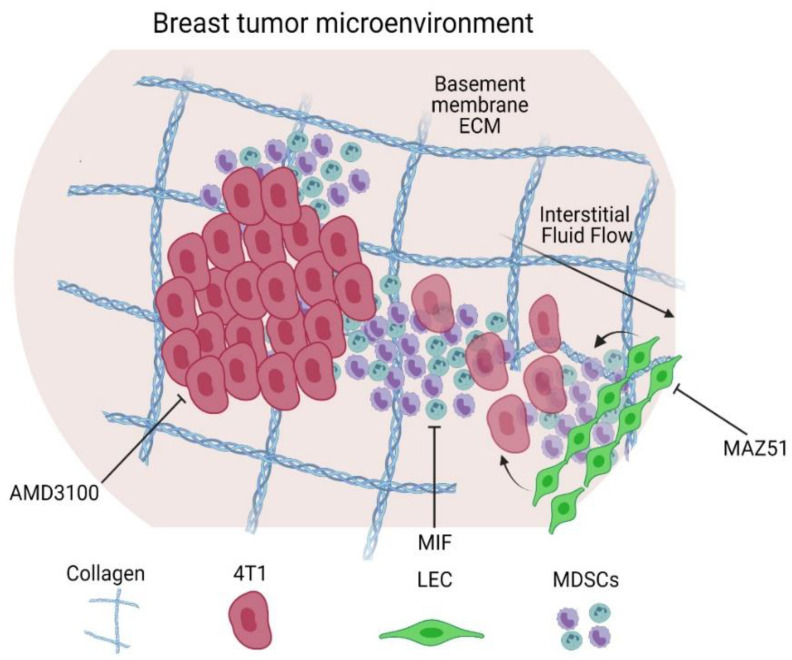
Schematic of invasion of LEC-4T1-MDSC crosstalk in the basement membrane extracellular matrix and inhibition under interstitial fluid flow. A straight arrow indicates interstitial fluid flow. Curved arrows indicate activation or interaction. Lines with stops indicate inhibition. (Created with BioRender.com. Accessed November 2021.)

In this study, we characterized migration of MDSCs within a 3D cell culture model designed to mimic some elements of the breast tumor microenvironment (Figure 6). We showed that GR1+ sorted splenocytes had reduced migration as compared to the unsorted population, including lower chemotactic index. One thought is that the beads alone are reducing the migration of the cells, and this can also be seen in the unsorted, bead-incubated MDSC population. However, other factors may be at play, including intercellular signaling, cell-specific migratory characteristics (i.e., GR1+ cells indeed migrate less), or other undetermined migratory effects. Regardless, caution should be considered when harvesting cells and assessing for migration as there can be distinctive effects of the harvest procedure alone.

In focusing on cells within the tumor microenvironment and the influence on MDSCs, we incorporated 4T1 mammary carcinoma cells and lymphatic endothelial cells in our model. In a recent study, MDA-MB-231 breast tumor cells and lymphatic–tropic derived cells secreted VEGF-C to prime LEC remodeling and subsequent chemokine/CXCR2 interaction to drive MDSC recruitment in a breast cancer orthotopic murine model [24], thus highlighting the importance of incorporating all of these cell types in our model. Here, we see that MDSCs do indeed chemotact towards lymphatic endothelial cells, but only in the context of interstitial flow. Since interstitial flow would be present in vivo, it is possible that these two stimuli do indeed work in concert to promote MDSC trafficking. However, we did not specifically look at this molecular modulator in MDSC migration, but our model could be useful in subsequent exploratory studies of these intercellular interactions. Further, we did not examine other elements of the breast tumor microenvironment that have been implicated in MDSCs, interstitial fluid flow, or lymphatic involvement. For example, fibroblasts are known to activate in response to interstitial fluid flow [57,58,59] and can contribute to immunosuppression [60]. Future models may choose to incorporate these cells in order to test these intercellular interactions.

To identify if there were specific direct mechanisms involved in MDSC migration, we inhibited key molecules in the function of MDSCs, tumor cells, and LECs. We observed that VEGFR3 inhibition had the greatest effect on suppressing both MDSC migration and 4T1 tumor cell migration. Tumor cells secrete VEGF-C as a ligand to VEGFR3 on the LECs for activation. This activation leads to tumor invasion through the lymphatics in breast cancer, thus, inhibition is critical to prevent tumor cell invasion [34]. Additionally, interstitial fluid flow, specifically transmural lymphatic flow, can lead to similar activation of VEGFR3. VEGFR3 activation in lymphatics can lead to a host of downstream signaling events that encourage cellular invasion, including chemokine secretion (CXCL12 or CCL21 for example), cellular adhesion upregulation (such as ICAM-1), and increased permeability. Most likely, a chemokine-mediated mechanism is responsible for the increased migration of MDSCs towards LECs; however, we did not determine the specific chemokine that is responsible in this study. Our results do align with previous studies demonstrating that LECs play a role in enhanced MDSC migration, and similar mechanisms may be at play [24].

It has been well-established that tumor cells secrete signaling cytokines to recruit MDSCs. However, our results showed that the presence of 4T1 tumor cells did not increase MDSC migration alone and required the presence of LECs and flow to promote overall MDSC migration. Potentially, the 4T1 cells are secreting chemokines that may co-opt MDSCs to modify the ECM, thereby limiting their migration on the timeframe of our experiments. Future work could examine longer timescales and analyze mediators and indicators of ECM modification to determine more complex behavioral interactions towards a more holistic understanding of LEC–tumor–MDSC crosstalk in the tumor microenvironment.

Our model offers both advantages and disadvantages for studying MDSCs in vitro. Studying MDSC migration characteristics in vitro in our 3D tissue engineered models was beneficial for examining function in a more controlled, easily observable system compared to in vivo studies, while allowing for recreation of part of the complex physiology seen in animal models. The disadvantages in our study were limitations with the methodology in harvesting MDSCs, including mechanical and enzymatic digestion of tissue and bead sorting for purification, which may have led to inadvertent differences in migratory behaviors [12,36]. In future studies, cell sorting through a robust panel of flow cytometry surface markers might be useful to achieve a purer population of MDSCs and identify sub-populations of MDSCs that may react differently in the tumor microenvironment. With each inoculation and harvest, our migration characteristics varied; thus, function can be impacted by methodology and timing. However, we were still able to gain novel insight on MDSC migration characteristics in our 3D tissue engineered breast cancer model and potential signaling molecules involved in tumor–lymphatic–MDSC crosstalk. Ultimately, our study demonstrated that MDSCs are sensitive to the tumor microenvironment in which they reside, particularly indicating that interstitial flow leads to enhanced migration when lymphatic endothelial cells are present and targeting of VEGFR3 may be a tractable mechanism to reduce both MDSC and tumor cell migration and, potentially, metastatic spread of disease.

## 5. Conclusions

This work evaluated MDSC function in a novel physiologically relevant 3D tissue engineered model, including the breast tumor microenvironment ECM, tumor and lymphatic cells, and interstitial fluid flow. We show that lymphatic cells increase migration of MDSCs and 4T1s in the presence of interstitial fluid flow. Moreover, pharmacological inhibition of molecules, important within the tumor microenvironment for cancer progression, highlighted the key role that VEGFR3 may play in tumor cell and MDSC migration. We propose a novel interaction of interstitial fluid flow and lymphatic involvement in MDSC migration in breast cancer. Our results provide initial insight on mechanisms that may be targeted for modulating the immune microenvironment.

## Data Availability

Data are available on request.

## Supplementary Materials

The following supporting information can be downloaded at: https://www.mdpi.com/article/10.3390/cancers14123008/s1. Figure S1: Magnetic beads used in separation reduced migratory characteristics of splenocytes, Figure S2: Migration characteristics are sensitive to harvest.
