# Supplementary figures and images for: Myeloid Derived Suppressor Cells Migrate in Response to Flow and Lymphatic Endothelial Cell Interaction in the Breast Tumor Microenvironment
Source: Cancers (Basel). 2022 Jun 18;14(12):3008. doi: 10.3390/cancers14123008 (PMC9221529; doi:10.3390/cancers14123008)

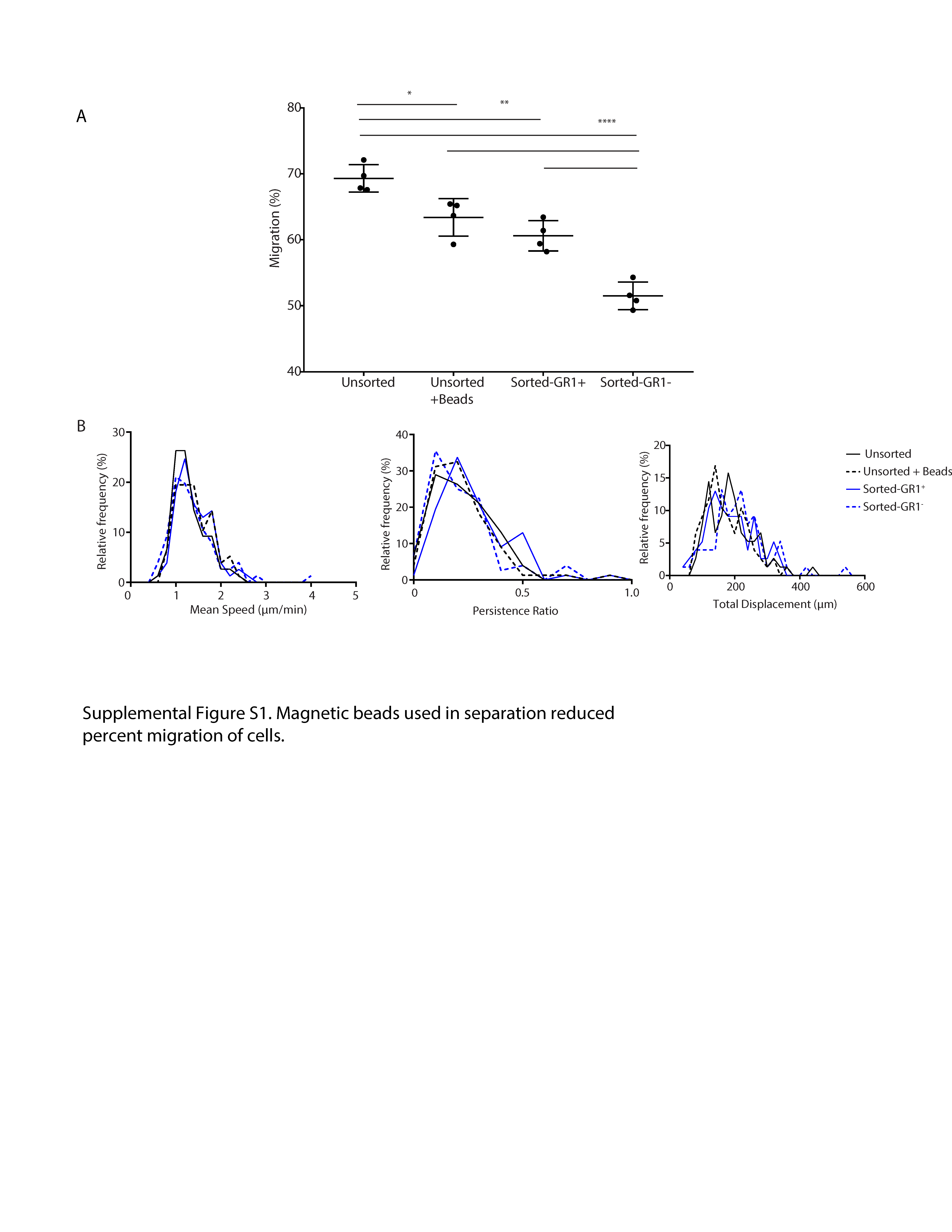

Supplement: Supplementary file 1 [file cancers-14-03008-s001.zip › Supplemental Figure S1.tif]

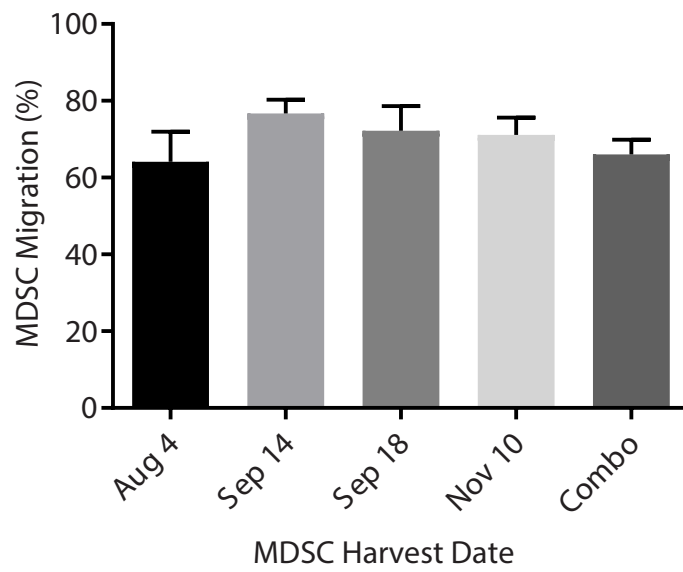

Supplemental Figure S2. Migration characteristics are sensitive to harvest.

Supplement: Supplementary file 1 [file cancers-14-03008-s001.zip › Supplemental Figure S2.pdf]

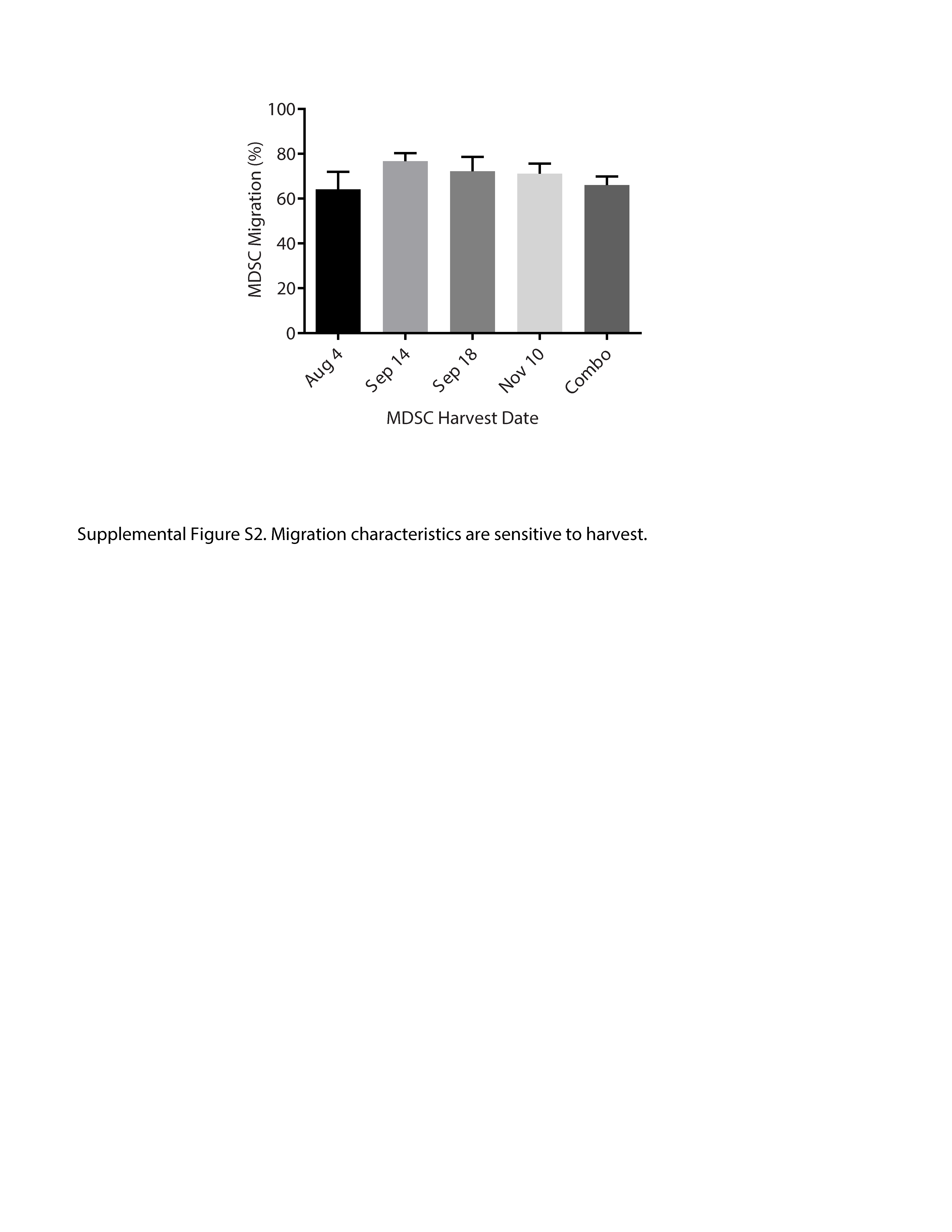

Supplement: Supplementary file 1 [file cancers-14-03008-s001.zip › Supplemental Figure S2.tif]
